# microRNA response elements-regulated TRAIL expression shows specific survival-suppressing activity on bladder cancer

**DOI:** 10.1186/1756-9966-32-10

**Published:** 2013-02-26

**Authors:** Youguang Zhao, Ying Li, Liang Wang, Hang Yang, Qingtang Wang, Haiyan Qi, Shadan Li, Peng Zhou, Ping Liang, Qiwu Wang, Xiaowei Li

**Affiliations:** 1 Department of Urology, General Hospital of Chengdu Military Area Command of Chinese PLA, Chengdu 610083, Sichuan Province, People’s Republic of China; 2 Department of Cardiology, General Hospital of Chengdu Military Area Command of PLA, Chengdu 610083Sichuan Province, People’s Republic of China; 3 Department of Urology, Qingdao Municipal Hospital, Qingdao 266300Shandong Province, People’s Republic of China

**Keywords:** Bladder cancer, Adenovirus, miRNA, Specificity, TRAIL

## Abstract

**Background:**

Bladder transitional cell carcinoma greatly threatens human health all over the world. Tumor necrosis factor-related apoptosis-inducing ligand (TRAIL) shows a strong apoptosis-inducing effect on a variety of cancer cells including bladder cancer. However, adenovirus-mediated TRAIL expression still showed cytotoxicity to normal cells mainly due to lack of tumor specificity.

**Methods:**

To solve the problem, we applied miRNA response elements (MREs) of *miR*-*1*, *miR*-*133* and *miR*-*218* to confer TRAIL expression with specificity to bladder cancer cells.

**Results:**

Expression of *miR*-*1*, *miR*-*133* and *miR*-*218* was greatly decreased in bladder cancer than normal bladder tissue. Luciferase assay showed that application of the 3 MREs was able to restrain exogenous gene expression to within bladder cancer cells. Subsequently, we constructed a recombinant adenovirus with TRAIL expression regulated by MREs of *miR*-*1*, *miR*-*133* and *miR*-*218*, namely Ad-TRAIL-MRE-1-133-218. qPCR, immunoblotting and ELISA assays demonstrated that Ad-TRAIL-MRE-1-133-218 expressed in bladder cancer cells, rather than normal bladder cells. The differential TRAIL expression also led to selective apoptosis-inducing and growth-inhibiting effect of Ad-TRAIL-MRE-1-133-218 on bladder cancers. Finally, bladder cancer xenograft in mouse models further confirmed that Ad-TRAIL-MRE-1-133-218 effectively suppressed the growth of bladder cancers.

**Conclusions:**

Collectively, we demonstrated that MREs-based TRAIL delivery into bladder cancer cells was feasible and efficient for cancer gene therapy.

## Background

Among the most common malignant cancers, bladder transitional cell carcinoma severely risks health of the people on the earth [1]. Downregulation of certain tumor suppressor genes was documented to largely contribute to initiation, progression, invasion and metastasis of bladder cancer [2]. Therefore, gene therapy is a reasonable strategy for bladder cancer treatment and many reports have confirmed its feasibility and effectiveness [3,4].

Tumor necrosis factor-related apoptosis-inducing ligand (TRAIL) has attracted much attention due to its specific induction of apoptosis in various types of cancer cells by binding death receptors and activating mitochondria-independent signal transduction pathway [5,6]. Like many other cancer types, adenovirus-mediated TRAIL therapy was well demonstrated to inhibit the survival of bladder cancer cells [7-12]. More intriguingly, extensive DR4 and DR5 expressions of bladder cancer in patients ensure its responsiveness to TRAIL in future clinical treatment [13].

Cytotoxicity to normal cells, however, seriously hurdles the clinical application of adenoviral vector for cancer gene therapy, since adenoviral vector lacks the ability to discriminate cancer and normal cells. To confer adenovirus with bladder cancer specificity, researchers developed many strategies including employing cancer-specific promoter. Although UP II promoter has been used to specifically drive TRAIL expression in bladder cancer cells, more novel strategies are needed to prevent the cytotoxicity of adenovirus-based gene therapy to normal cells [14-16].

Differential expression profile of miRNAs has been widely reported between bladder cancer and normal cells [17]. Decreased expression level of certain miRNAs allows the introduced genes specifically expressed in bladder cancer cells by inserting their miRNA response elements (MREs) following the opening reading frames. So far, no groups have tested the feasibility and effectiveness of this MREs-based strategy for bladder cancer-specific gene therapy.

Here, we intended to identify suitable MREs for bladder cancer specific adenovirus-mediated TRAIL expression from the miRNAs with downregulated expression in bladder cancer, including miR-1 [18-21], miR-99a [22], miR-100 [23], miR-101 [24,25], miR-125b [23,26,27], miR-133a [18,20,21,23,28-30], miR-143 [22,23,31-33], miR-145 [21,23,29-31,34], miR-195-5p [35], miR-199a-3p [36], miR-200 [37,38], miR-203 [39,40], miR-205 [37], miR-218 [21,41], miR-490-5p [42], miR-493 [43], miR-517a [44], miR-574-3p [45], miR-1826 [46] and let-7c [42].

## Methods

### Primary culture

We employed primary cultures derived from bladder transitional carcinoma and normal bladder mucosal cells (BMC) in this study. For the culturing of bladder cancer, the samples were obtained with written informed consent from all patients according to protocols approved by Ethical Review Board in General Hospital of Chengdu Military Area Command of Chinese PLA (Chengdu, China). All patients underwent surgical resection of bladder carcinoma at Department of Urology, General Hospital of Chengdu Military Area Command of Chinese PLA (Chengdu, China). Bladder cancer samples were sheared into small pieces, followed by mechanical manipulation to obtain single cell suspension. The primary cultures were maintained in DMEM supplemented with 15% FBS.

For primary BMC culture, the samples were obtained from 8 patients that underwent cystoscopic examination of asymptomatic haematuria (The biopsies were not malignant revealed by histopathological results). The previously described procedures that have been approved by Ethical Review Board in General Hospital of Chengdu Military Area Command of Chinese PLA (Chengdu, China) was followed to establish the primary BMC culture [47]. The BMCs were immortalized using adenoviral vector, Adeno-SV40 (Applied Biological Materials Inc., Canada), according to the manufacturer’s instructions. All the patients approved the application of their samples for this study.

### Construction of adenoviral vectors

Ad-EGFP and Ad-TRAIL were preserved in our laboratory. We constructed Ad-TRAIL-MRE-1-133-218 as follows. A DNA fragment was synthesized (5′-ACAAACACCACATTCCAACAAACACCACATTCC.

**Figure 1:**
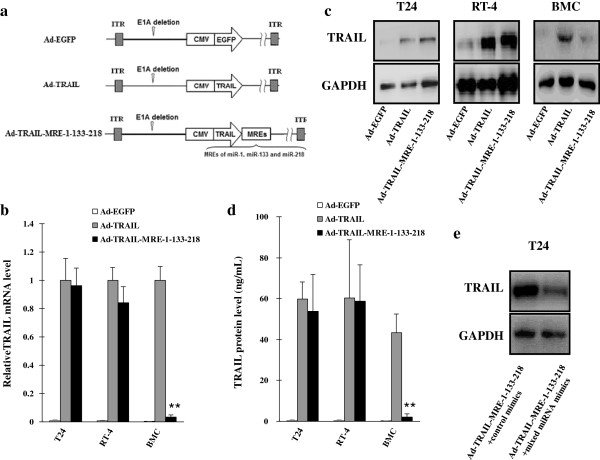
**MREs of miR**-**1**, **miR**-**133 and miR**-**218 enabled adenovirus**-**mediated adenoviral vector to express TRAIL with bladder cancer specificity.** (**a**) Illustration was shown of the structures of the involved adenoviral vectors. Ad-TRAIL-MRE-1-133-218 contained MREs of miR-1, miR-133 and miR-218 that were inserted immediately following TRAIL gene. ITR: inverse terminal region. (**b**) qPCR assay was performed to detect TRAIL mRNA expression. TRAIL mRNA levels in Ad-TRAIL cells were selected as standards and GAPDH was selected as endogenous reference. Means ± SEM of three independent experiments were shown. (**c**) TRAIL protein level was also determined in T24 and RT-4 bladder cells as well as BMCs infected with different adenoviruses by immunoblotting. GAPDH was selected as endogenous reference. (**d**) TRAIL protein level was also evaluated in the same cells infected with the indicated adenoviruses by ELISA assay. Means ± SEM of three independent experiments were shown. (**e**) T24 cells were treated with both 10 MOI of Ad-TRAIL-MRE-1-133-218 and mixed mimics of *miR*-*1*, *miR*-*133* and *miR*-*218* (100 nM for each) or control mimics (300 nM). 48 h later, TRAIL expression was tested by immunoblotting assay. GAPDH was selected as endogenous reference.

AACAAACACCGGACCAAAACAAACACCGGACCAAAACAAACACCAAGCACAAACAAACACCAAGCACAA-3′), which contained two copies of miR-1 MREs, two copies of miR-133 MREs and two copies of miR-218 MREs. This fragment was released from the temporary vector by *EcoRV* and then inserted into pShuttle-CMV-TRAIL at the same site, generating pShuttle-CMV-TRAIL-MRE-1-133-218. This plasmid was subsequently cotransfected into HEK-293 cells with pAdEasy. After plague purification for three times and PCR-based identification, adenoviruses were harvested and then purified with the CsCl gradient centrifugation. The involved adenoviruses were titrated with TCID_50_ method on HEK-293 cells and represented as plaque-forming units per milliliter (pfu/ml) [48]. The adenovirus was designated as Ad5-TRAIL-MRE-1-133-218. The structures of these adenoviruses were shown in Figure 1a.

### Cell line cultures

Human bladder transitional cell carcinoma cell line T24 and RT-4 were both purchased from the American Type Culture Collection (Manassas, VA) and were grown in McCoy’s 5a Medium Modified (Life Technologies, Rockville, MD) supplemented with 10% (v/v) fetal bovine serum (Life Technologies, Rockville, MD). Human endothelial cells HUV-EC-C and normal liver cells L-02 were obtained from Shanghai Cell Collection (Shanghai, China). HUV-EC-C and L-02 cells were cultured using DMEM media supplemented with 10% (v/v) fetal bovine serum. All media was supplemented with 4 mM glutamine, 100 units/mL penicillin and 100 μg/ml streptomycin. All cells in this experiment were cultured under a 5% CO_2_ and humidified atmosphere at 37°C.

### Quantitative PCR (qPCR)

Total RNA was extracted from 14 bladder cancer samples with Trizol solution (Sigma-Aldrich, MO) and pooled as one group for subsequent experiments. Another pool of RNA was also obtained from 8 normal bladder mucosal tissues according to the same protocol. Also, T24, RT-4, HUV-EC-C and L-02 cells were processed for extracting RNA with Trizol solution. Reverse transcription reaction was subsequently performed with TaqMan® MicroRNA Reverse Transcription Kit (Applied Biosystems) according to the manufacturer’s instructions. qPCR was finally performed with TaqMan® 2 × Universal PCR Master Mix (Applied Biosystems) on CFX96™ Real-Time PCR Detection System (Bio-Rad Laboratories, CA) supplied with analytical software.

4 × 10^4^ cells were cultured in each well of 6-well plates. TRAIL mRNA abundance was determined in Ad-TRAIL-MRE-1-133-218-infected cells after treated with 10 MOI of adenoviruses. After 48h, cells were lysed for RNA extraction and then inversely transcribed into cDNAs with Rever Tra Ace qPCR RT Kit (Toyobo, Japan) according to the manufacturer’s instructions. qPCR was performed with SYBR premix Ex Taq (TaKaRa) on CFX96™ Real-Time PCR Detection System (Bio-Rad Laboratories, CA) supplied with analytical software.

### Immunoblotting assay

Protein in adenovirus-infected cells was quantified with immunoblotting assay. 3.5 × 10^5^ cells were cultured in each well of 6-well plates. 10 MOI of adenoviruses were added to cell cultures. Proteins were lyzed with M-PER® Mammalian Protein Extraction Reagent (Thermo Scientific, IL) after 48 h, separated using polyacrylamide gel electrophoresis and transferred onto 0.45 μm nitrocellulose membranes. 5% fat-free dry milk was used for blocking. The membrane was then incubated with specific primary antibodies for 6h. The membrane was incubated with corresponding secondary antibody and then with SuperSignal West Dura Extended Duration Substrate (Thermo Scientific, IL).

### TRAIL determination by ELISA assay

We performed ELISA assay to evaluate the secreted TRAIL protein in media. Briefly, 3.5 × 10^5^ cells were cultured in each well of 6-well plates. 10 MOI of adenoviruses were added to cell media. After 48h, two-antibody sandwich ELISA was applied to determine human TRAIL expression level in the supernatant of cells. The involved antibodies are monoclonal mouse anti-human TRAIL antibody (R&D Systems), peroxidase-conjugated rabbit anti-goat IgG (H&L) and goat anti-human TRAIL antibody (R&D Systems). The absorbance was assessed at a 450 nm wavelength.

### miRNA mimics treatment

*miR*-*1*, *miR*-*133*, *miR*-*218* and control mimics were synthesized by GenePharma (Shanghai, China). T24 and RT-4 cells were transfected with 300 nM control mimic or the mixture of 100 nM *miR*-*1*, 100 nM *miR*-*133* and 100 nM *miR*-*218*.

### FACS analysis on apoptotic rates

3.5 × 10^5^ cells were cultured in each well of 6-well plates. After 24h, the cells were infected with adenoviruses of 10 MOI. After 48h, the cells were stained with Annexin V-PE Apoptosis Detection Kit (Biovision, CA) based on the manufacturer’s instructions. The percentages of apoptotic cells were examined with FACS analysis.

### Luciferase assay

The synthesized DNA constructs, which contains two copies of indicated MREs, were inserted into the *XhoI* and *NotI* sites of psiCheck2 vectors (Promega, WI) to construct recombinant luciferase reporter (psiCheck2-*). The involved MREs sequences in our study were described in detail in Table 1.

**Table 1:** MiRNA response elements (MREs) for bladder cancer-specific downregulated miRNAs

**miRNA**	**primer sequences**
*miR-1*	Forward: 5′-TCGAGACAAACACCACATTCCAACAAACACCACATTCCAACAAACACCGC-3′
	Reverse: 5′-GGCCGCGGTGTTTGTTGGAATGTGGTGTTTGTTGGAATGTGGTGTTTGTC-3′
*miR-99a*	Forward: 5′-TCGAGACAAACACCTACGGGTACAAACACCTACGGGTACAAACACCGC-3′
	Reverse: 5′-GGCCGCGGTGTTTGTACCCGTAGGTGTTTGTACCCGTAGGTGTTTGTC-3′
*miR-101*	Forward: 5′-TCGAGACAAACACCGTACTGTACAAACACCGTACTGTACAAACACCGC-3′
	Reverse: 5′-GGCCGCGGTGTTTGTACAGTACGGTGTTTGTACAGTACGGTGTTTGTC-3′
*miR-133*	Forward: 5′-TCGAGACAAACACCGGACCAAAACAAACACCGGACCAAAACAAACACCGC-3′
	Reverse: 5′-GGCCGCGGTGTTTGTTTTGGTCCGGTGTTTGTTTTGGTCCGGTGTTTGTC-3′
*miR-218*	Forward: 5′-TCGAGACAAACACCAAGCACAAACAAACACCAAGCACAAACAAACACCGC-3′
	Reverse: 5′-GGCCGCGGTGTTTGTTTGTGCTTGGTGTTTGTTTGTGCTTGGTGTTTGTC-3′
*miR-490-5p*	Forward: 5′-TCGAGACAAACACCATCCATGACAAACACCATCCATGACAAACACCGC-3′
	Reverse: 5′-GGCCGCGGTGTTTGTCATGGATGGTGTTTGTCATGGATGGTGTTTGTC-3′
*miR-493*	Forward: 5′-TCGAGACAAACACCACCTTCAACAAACACCACCTTCAACAAACACCGC-3′
	Reverse: 5′-GGCCGCGGTGTTTGTTGAAGGTGGTGTTTGTTGAAGGTGGTGTTTGTC-3′
*miR-517a*	Forward: 5′-TCGAGACAAACACCTGCACGAACAAACACCTGCACGAACAAACACCGC-3′
	Reverse: 5′-GGCCGCGGTGTTTGTTCGTGCAGGTGTTTGTTCGTGCAGGTGTTTGTC-3′

The underscored sequences indicated MREs of *miR-1, miR-99a, miR-101, miR-133 and miR-218, miR-490-5p, miR-493* and *miR-517a*.

4 × 10^4^ cells were cultured in each well of 24-well plates. After transfecting T24, RT-4 and BMCs with the above plasmids, cells were processed with lysis buffer, and subsequently, luciferase activities were assessed with the Dual-Luciferase reporter system (Promega, WI) according to the manufacturers’ instructions.

### Cell viability assay

1 × 10^4^ T24 and RT-4 cells, 1.5 × 10^4^ primary bladder cancer cells or 2 × 10^4^ BMCs were cultured in each well of 96-well plates. Adenoviruses of indicated MOIs were added to cell cultures. After 6d, 50 μl of MTT (1 mg/ml) was added, and 4 h later, MTT-containing media was replaced with 150 μl of DMSO. The spectrophotometric absorbance was assessed on a model 550 microplate reader (Bio-Rad Laboratories, Hercules, CA) at 570 nm with a reference wavelength of 655 nm. Cell viability = absorbance value of infected cells / absorbance value of uninfected control cells.

### Animal experiments

Procedures for animal experiments were all approved by the Committee on the Use and Care on Animals in Qingdao Municipal Hospital (Qingdao, China).

2×10^6^ T24 cells were inoculated at the left flanks of 5-week-old female BALB/c nude mice (Institute of Animal Center, Chinese Academy of Sciences, Shanghai, China). When tumors reached 7–9 mm in diameter, 24 mice were equally assigned into 4 groups (n=6). 100 μL of PBS with or without 2×10^8^ pfu of Ad-EGFP, Ad-TRAIL and Ad-TRAIL-MRE-1-133-218 was directly administrated into tumors by injection, respectively. The administrations were performed every other day for five times with a total dosage of 1×10^9^ pfu of adenoviruses.

T-24 cancer xenograft was established by incubating 1.5×10^6^ cells at the right flanks of 5-week-old female BALB/c nude mice. 24 mice were equally divided into 4 groups (n=6). The doses of used adenoviruses and injection procedures were the same as those on T24 tumor xenograft.

We periodically measured tumor diameter using calipers. Tumor volume (mm^3^) = maximal length (mm) × perpendicular width (mm) ^2^ / 2.

### Liver function evaluation

To evaluate the hepatoxicity induced by adenovirus treatment, BALB/c mice (n=5) were intravenously injected with 1×10^9^ pfu of indicated adenoviruses every other day for five times. On day 11, their blood (600 mL/mice) was harvested by cardiac puncture, followed by being incubated with 12 U of heparin. Alanine aminotransferase (ALT) levels in blood were detected at the Clinical Laboratory, Qingdao Manucipal Hospital (Qingdao, China).

### Histological staining

On day 7 after adenovirus injection, one mouse was sacrificed from each group and its tumor, brain and liver were collected and fixed according to the routine procedures. Histological staining was then performed on formalin-fixed, paraffin-embedded tumor, brain and liver tissue sections using the streptavidinbiotin peroxidase complex method. Anti-TRAIL antibody (Santa Cruz Biotechnology, CA) was used to specifically recognize TRAIL protein. The sections were finally counterstained with hematoxylin.

### Statistical analysis

The statistical tests in this manuscript were two-tailed student’s t-test. Differences were considered as statistically significant (*) when P < 0.05 and statistically very significant (**) when P < 0.01.

## Results

### The expression levels of 8 miRNAs were greatly reduced in bladder cancer cells

**Figure 2:**
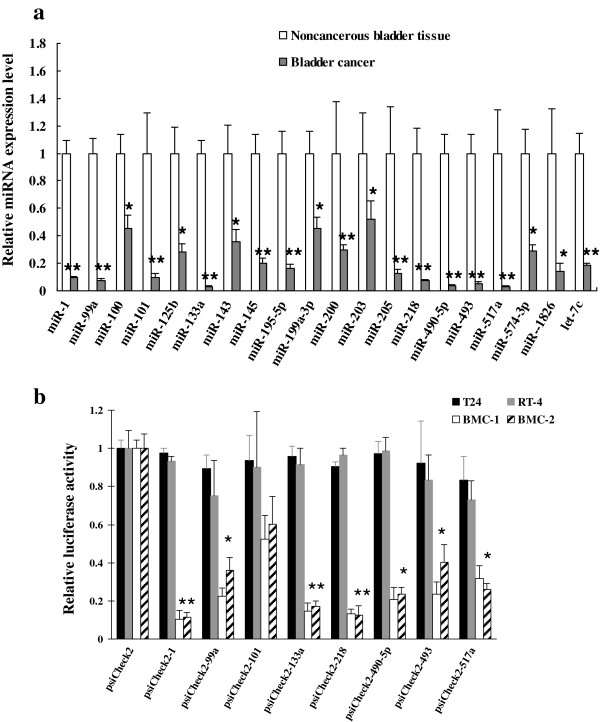
**MREs**-**regulated expression of exogenous gene in bladder cancer cells.** (**a**) Expression of different miRNAs was detected in the pooled 14 bladder cancer and 8 normal bladder mucosal tissues. miRNA level in noncancerous bladder tissue was regarded as standard and U6 was selected as endogenous reference. Means ± SEM of three independent experiments were shown. (**b**) LuciferBMCase activity was quantified in T24 and RT-4 bladder cells as well as s that were transfected with luciferase reporter plasmids. The luciferase activity in these cells transfected with psiCheck2 was used as standard. Means ± SEM of three independent experiments were shown.

To experimentally identify downregulated miRNAs in cancerous tissues derived from bladder epithelium, we studied miRNA expression profiles in 14 bladder cancer samples. qPCR assay showed that expression levels of all the tested miRNAs were decreased in bladder cancer cells in comparison with 8 noncancerous bladder tissue. Among them, *miR*-*1*, *miR*-*99a*, *miR*-*101*, *miR*-*133a*, *miR*-*218*, *miR*-*490*-*5p*, *miR*-*493 and miR*-*517a* had reduction of greater than 90% in their expression level (P<0.01) (Figure 2a). Also, we detected the expression levels of *miR*-*1*, *miR*-*99a*, *miR*-*101*, *miR*-*133a*, *miR*-*218*, *miR*-*490*-*5p*, *miR*-*493 and miR*-*517a* in T24 and RT-4 bladder cancer cell lines. Consistently, their levels were reduced in the tested cell lines (Additional file 1: Figure S1). The differential expression profile of miRNAs ensured the possibility of utilizing these miRNAs to specifically express genes of interests in bladder cancer cells.

### Application of MREs of miR-1, miR-133 and miR-218 restrained exogenous gene expression within bladder cancer cells

To assess if MREs of *miR*-*1*, *miR*-*99a*, *miR*-*101*, *miR*-*133a*, *miR*-*218*, *miR*-*490*-*5p*, *miR*-*493 and miR*-*517a* could be used for bladder cancer-specific delivery of exogenous genes, we constructed a series of reporter plasmids containing luciferase regulated by their MREs. The data revealed that luciferase expression was only slightly affected in bladder cancer cells transfected with the reporter plasmids that were regulated by MREs of *miR*-*1*, *miR*-*101*, *miR*-*133a*, *miR*-*218* and *miR*-*490*-*5p* (Figure 2b). Furthermore, inhibitory effect on luciferase expression was greater than 80% in bladder mucosal cells (BMCs) when MREs of *miR*-*1*, *miR*-*133a* and *miR*-*218* were used (P<0.01) (Figure 2b). Furthermore, HUV-EC-C and normal liver cells L-02 have been shown to have much higher expression level of *miR*-*1*, *miR*-*133a* and *miR*-*218* than bladder cancer samples (Additional file 2: Figure S2).

### Bladder cancer-specific expression of TRAIL genes was achieved by employing MREs of miR-1, miR-133 and miR-218

To confirm if combined application of MREs of *miR*-*1*, *miR*-*133* and *miR*-*218* conferred TRAIL expression with bladder cancer specificity, we simultaneously inserted the 3 MREs immediately following TRAIL-encoding open reading frame on adenoviral vectors (Figure 1a). qPCR assay showed that the modified adenovirus, Ad-TRAIL-MRE-1-133-218, had a similar level of TRAIL gene to that of Ad-TRAIL in bladder cancer while TRAIL expression was greatly suppressed in Ad-TRAIL-MRE-1-133-218-infected BMC (Figure 1b). Immunoblotting and ELISA assays also confirmed that Ad-TRAIL-MRE-1-133-218 infection resulted in TRAIL expression with a comparative level with Ad-TRAIL, but almost no TRAIL expression was detected in normal bladder mucosal cells infected with Ad-TRAIL-MRE-1-133-218 (Figure 1c and d).

To confirm MRE-regulated TRAIL expression was dependant on the level of corresponding miRNAs, Ad-TRAIL-MRE-1-133-218-infected T24 cells were treated with mixed mimics of *miR*-*1*, *miR*-*133* and *miR*-*218*. Elevated expression level of these miRNAs led to a great reduction in TRAIL expression in bladder cancer cells (Figure 1e).

The above results verified that simultaneous application of MREs of *miR*-*1*, *miR*-*133* and *miR*-*218* conferred adenovirus-mediated TRAIL expression with bladder cancer specificity.

### MREs-regulated adenovirus-mediated TRAIL expression specifically activated extrinsic apoptotic pathway in bladder cancer cells

**Figure 3:**
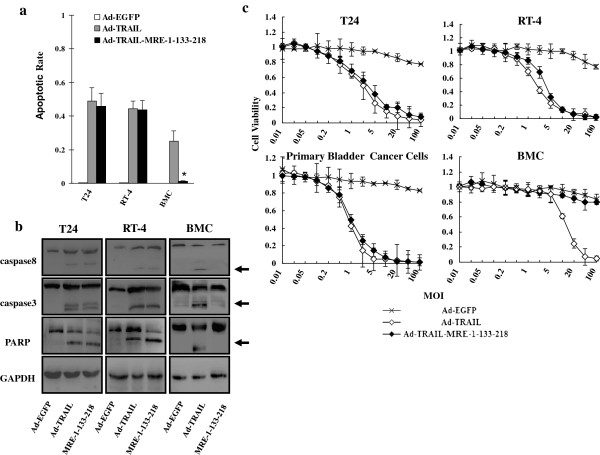
**Anti**-**tumor capacity of Ad**-**TRAIL**-**MRE**-**1**-**133**-**218 on bladder cancer cells with no significant cytotoxicity to normal cells.** (**a**) Apoptosis was detected in the indicated cells by FACS analysis on Annexin V expression. Means ± SEM of three independent experiments were shown. (**b**) Cleavages of caspase 3, caspase 8 and PARP were determined by immunoblotting assay. Arrows indicated the cleaved fragments of these proteins. GAPDH was selected as endogenous reference. (**c**) Viability of different cells was determined after the indicated adenoviruses were applied. The absorptive values of cells without adenovirus infection were used as standards. Means ± SEM of three independent experiments were shown.

As a well-known proapoptotic protein, TRAIL induced apoptosis in a variety of cancer types through activating extrinsic apoptotic pathway. Therefore, we investigated if normal bladder mucosal cells evaded the apoptosis induced by TRAIL expression by Ad-TRAIL-MRE-1-133-218. FACS analysis showed that apoptosis took place selectively in bladder cancer cells, rather than normal bladder cells, when Ad-TRAIL-MRE-1-133-218 was employed. In contrast, Ad-TRAIL induced apoptosis both in bladder cancerous and normal cells. In addition, there was no significant difference in apoptotic rate between Ad-TRAIL- and Ad-TRAIL-MRE-1-133-218-treated bladder cancer cells, suggesting no impairment of apoptosis-inducing capacity caused by this modification (Figure 3a).

We subsequently examined the activation of extrinsic apoptosis pathway in T24, RT-4 and BMC cells by immunoblotting assay. The data showed that caspase-8 was cleaved in Ad-TRAIL and Ad-TRAIL-MRE-1-133-218-infected bladder cancer cells as well as Ad-TRAIL-infected BMCs. However, this cleavage did not take place in Ad5-TRAIL-MRE-1-133-218-treated normal bladder mucosal cells (Figure 3b). Similarly, cleavages of caspase-3 and PARP proteins were also observed in the same patterns as caspase-8, suggesting extrinsic apoptotic pathway was selectively activated in bladder cancer cells when Ad5-TRAIL-MRE-1-133-218 was used (Figure 3b).

### Ad-TRAIL-MRE-1-133-218 decreased the survival of bladder cancer cells rather than normal bladder mucosal cells

We next investigated the viability of bladder cancer cells and BMCs with MTT assay, when Ad-EGFP, Ad-TRAIL and Ad-TRAIL-MRE-1-133-218 were added to the indicated cell cultures. The data revealed that Ad-TRAIL-MRE-1-133-218 had a comparative tumor-suppressing capacity on T24 and RT-4 bladder cancer cells as well as primary bladder carcinoma cells with Ad-TRAIL (Figure 3c). However, Ad-TRAIL had cytotoxicity to both cancerous and normal bladder cells. In contrast, administration of Ad-TRAIL-MRE-1-133-218 did not affect the survival of BMCs.

Collectively, we proved that Ad-TRAIL-MRE-1-133-218 inhibited the viability of bladder cancer cells without significant cytotoxicity to normal cells.

### Ad-TRAIL-MRE-1-133-218 suppressed the growth of bladder cancer xenograft in mouse models

**Figure 4:**
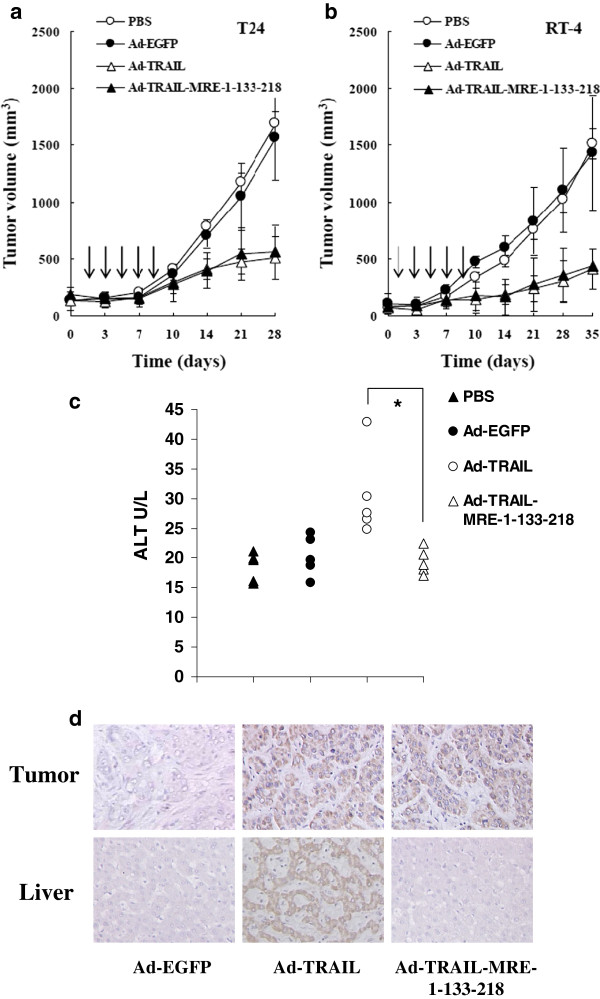
**Ad**-**TRAIL**-**MRE**-**1**-**133**-**218 suppressed the growth of bladder xenograft in mouse models.** (**a**) T24 bladder cancer xenograft was established by subcutaneously injecting 2×10^6^ cells into left flanks of female BALB/c nude mice. 1×10^9^ pfu of different adenoviruses were treated and the tumor volumes were periodically measured. Means ± SEM of tumor sizes were shown. The arrows indicated time-points of adenovirus injection. (**b**) RT-4 xenograft was established by subcutaneously injecting 1.5×10^6^ cell into right flanks of female BALB/c nude mice. 1×10^9^ pfu of different adenoviruses were treated and the tumor volumes were periodically measured. Means ± SEM of tumor sizes were shown. The arrows indicated time-points of adenovirus injection. (**c**) BALB/c nude mice (n=5) were intravenously injected with 1×10^9^ pfu of different adenoviruses every other days for five times. On day 11, their blood was harvested for the measurement of ALT levels. Means ± SEM of ALT serum levels were shown. (**d**) Histological staining was performed to detect TRAIL expression in tumor and liver section from the tumor-bearing mice after treatment of Ad-EGFP, Ad-TRAIL and Ad-TRAIL-MRE-1-133-218. The representative images were shown (×200).

Next, we intended to further investigate the suppressive action of Ad-TRAIL-MRE-1-133-218 on bladder cancer xenograft using mouse models. T24 and RT-4 bladder cancer cells were used to establish the tumor xenografts. We periodically recorded the growth of these bladder cancer xenografts when Ad-EGFP, Ad-TRAIL and Ad-TRAIL-MRE-1-133-218 were administered. The data demonstrated that Ad-TRAIL and Ad-TRAIL-MRE-1-133-218 had a similar growth-inhibiting effect on both T24 and RT-4 bladder cancers (Figure 4a and b). The animal experiments consistently demonstrated that MREs-regulated adenovirus-mediated TRAIL expression had a strong tumor-suppressing effect on bladder cancer.

To test the side effect induced by these adenoviruses, we injected Ad-EGFP, Ad-TRAIL and Ad-TRAIL-MRE-1-133-218 into BALB/c mice. On day 11, their blood was collected and assayed for ALT level in serum. Ad-TRAIL treatment was found to cause an elevated level of serum ALT in mice. In contrast, Ad-TRAIL-MRE-1-133-218 did not significantly change the ALT level in the blood of mice, showing no cytotoxicity to liver cells (Figure 4c).

Also, TRAIL expression was evaluated in the tumor and liver sections from the T24 tumor-bearing mice that received the injection of Ad-EGFP, Ad-TRAIL and Ad-TRAIL-MRE-1-133-218. The histological staining showed that Ad-TRAIL-MRE-1-133-218 treatment resulted in high expression of TRAIL in tumors as Ad-TRAIL infection (Figure 4d). Importantly, TRAIL expression was not detected in liver section from Ad-TRAIL-MRE-1-133-218-treated group, whereas Ad-TRAIL-infected mice had an extensive TRAIL expression in their livers (Figure 4d).

## Discussion

In this study, we experimentally confirmed expression profiles of 20 miRNAs in bladder cancer and corresponding noncancerous bladder tissues. qPCR assay showed that all of them had lower abundance in bladder cancer in comparison with normal bladder tissue. Our results were in accordance with previous reports from other research groups. The differential expression level of these miRNAs made it feasible that their MREs can be utilized to control TRAIL expression specifically in bladder cancer cells.

Luciferase reporter assays showed that *miR*-*1*, *miR*-*99a*, *miR*-*101*, *miR*-*133a*, *miR*-*218*, *miR*-*490*-*5p*, *miR*-*493* and *miR*-*517a* only had limited suppressive effect on luciferase expression in bladder cancer cells when their MREs were applied. Further investigation indicated that MREs of *miR*-*1*, *miR*-*133a* and *miR*-*218* inhibited luciferase expression in normal bladder cells. Therefore, MREs of *miR*-*1*, *miR*-*133a* and *miR*-*218* were believed to prevent exogenous gene expression from normal bladder mucosal cells without affecting its expression in bladder cancer cells.

UPII promoter has been utilized for specific TRAIL expression in bladder cancer cells. However, gene expression controlled by this promoter is not strictly bladder cancer-specific, due to the remaining activity of UPII promoter in normal bladder mucosal cells [49]. Therefore, other strategies should be developed for preventing TRAIL expression from normal bladder cells. We employed multidisciplinary approaches to prove that TRAIL expression was greatly inhibited in Ad-TRAIL-MRE-1-133-218-infected normal bladder epithelial cells. These data demonstrated this recombinant adenovirus as a vehicle for TRAIL expression with a high bladder cancer-specificity.

As expected, Ad-TRAIL-MRE-1-133-218 induced extrinsic pathway-mediated apoptosis in bladder cancer cells, rather than normal bladder mucosal cells. Subsequent cell viability assay and animal experiments showed that Ad-TRAIL-MRE-1-133-218 greatly suppressed the growth of bladder cancer. More importantly, survival of normal bladder epithelial cells was almost not affected by Ad-TRAIL-MRE-1-133-218, suggesting biosafety of this MREs-regulated TRAIL-expressing adenoviral vector.

To further improve the biosafety of the adenoviral vector expressing TRAIL, other MREs should also be applied to suppress the undesirable exogenous gene expression in normal tissue, such as liver. *miR*-*122* has been extensively reported to be highly expressed in normal hepatic cells and downregulated in hepatocellular carcinoma, and thus, its MRE can be utilized to prevent cytotoxicity from liver cells [50].

TRAIL has been demonstrated as a potent anti-tumor cytokine in our study. Other therapeutic cytokines also act as candidates for cancer gene therapy, especially the natural inhibitors against signaling pathway that is critical for cancer progression. For example, DKK1 has been shown to suppress the gastric cancer progression by inhibiting WNT/β-catenin pathway [51]. Our novel MRE-regulated adenoviral vector is believed to be a suitable expression vehicle for these inhibitors with high bladder cancer specificity.

## Conclusions

We generated a bladder cancer-specific adenoviral vector that expressed TRAIL based on MREs of miRNAs whose levels were reduced in bladder cancer. The anti-tumor capacity and biosafety of this new adenoviral vector was proved by a series of experimental approaches. We proposed that the MREs-targeted adenovirus is a promising tool for gene therapy against bladder cancer.

## Competing interests

The authors declare that they have no competing interests.

## Authors’ contributions

YZ and YL designed the study. YZ, YL, LW, HY, QW, HQ, SL, PZ, PL, QW and XL performed the experiments. YZ and YL drafted the manuscript. YZ supervised the experimental work. All authors read and approved the final manuscript.

## Supplementary Material

Additional file 1: Figure S1 Etoptic miRNA expression profile of T24 and RT-4 cells. Expression of *miR*-*1*, *miR*-*99a*, *miR*-*101*, *miR*-*133a*, *miR*-*218*, *miR*-*490*-*5p*, *miR*-*493 and miR*-*517a* were detected in T24 and RT-4 cells. miRNA level in noncancerous bladder tissue was regarded as standard and U6 was selected as endogenous reference. Means ± SEM of three independent experiments were shown. (DOC 39 kb) Click here for file

Additional file 2: Figure S2 Differential expression levels of *miR*-*1*, *miR*-*133a* and *miR*-*218* between normal cells and bladder cancer Expression of *miR*-*1*, *miR*-*133a* and *miR*-*218* were detected in HUV-EC-C and L-02 cells. miRNA level in HUV-EC-C cells was regarded as standard and U6 was selected as endogenous reference. Means ± SEM of three independent experiments were shown. (PPT 115 kb) Click here for file
