# Supplementary figures and images for: microRNA response elements-regulated TRAIL expression shows specific survival-suppressing activity on bladder cancer
Source: J Exp Clin Cancer Res. 2013 Feb 26;32(1):10. doi: 10.1186/1756-9966-32-10 (PMC3764979; doi:10.1186/1756-9966-32-10)

## Slide 1
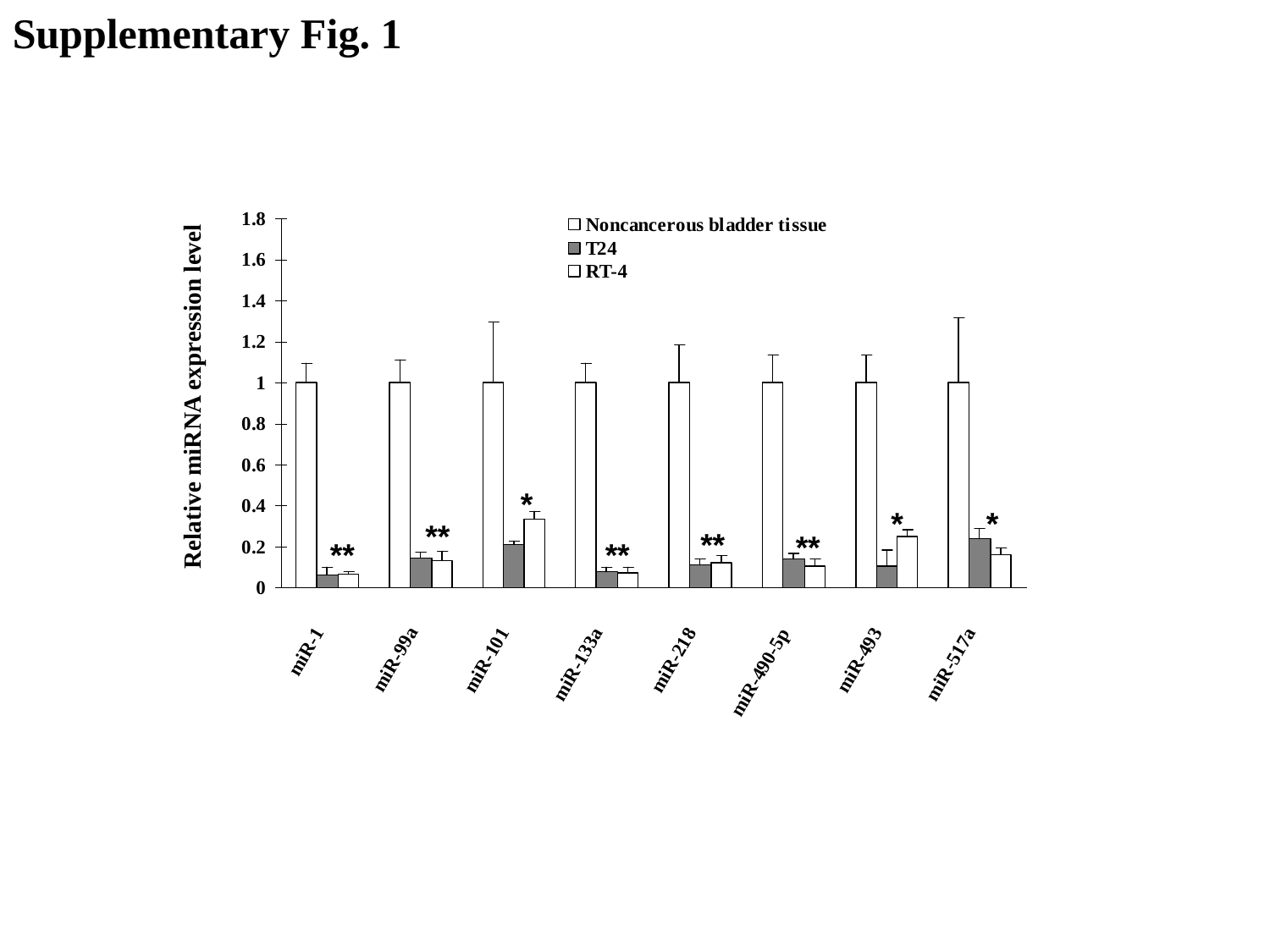

Supplementary Fig. 1
Relative miRNA expression level
*
*
*
**
**
**
**
**

Supplement: Additional file 1: Figure S1 — Etoptic miRNA expression profile of T24 and RT-4 cells. Expression of miR-1, miR-99a, miR-101, miR-133a, miR-218, miR-490-5p, miR-493 and miR-517a were detected in T24 and RT-4 cells. miRNA level in noncancerous bladder tissue was regarded as standard and U6 was selected as endogenous reference. Means ± SEM of three independent experiments were shown. (DOC 39 kb) [file 1756-9966-32-10-S1.ppt]

## Slide 1
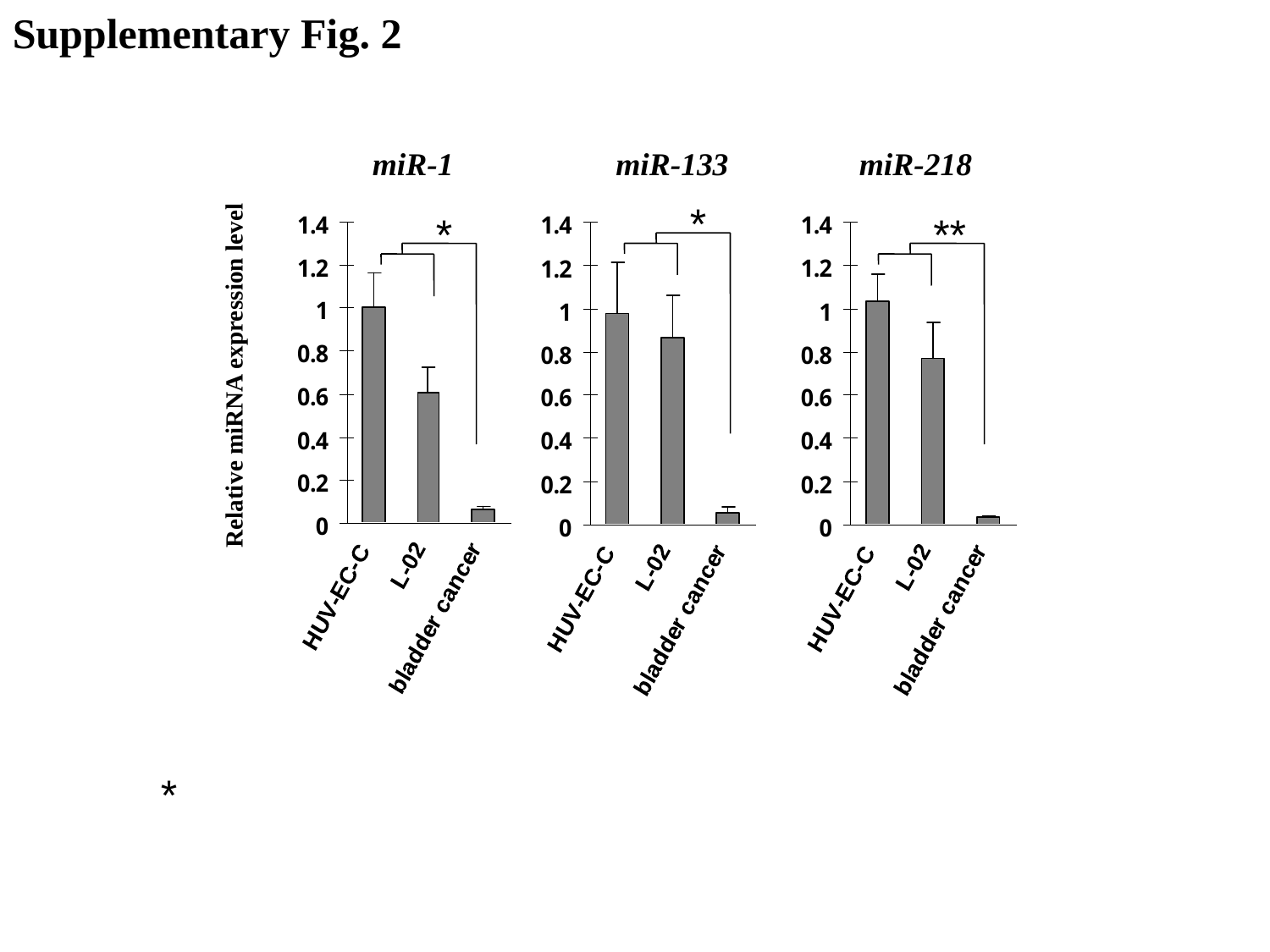

Supplementary Fig. 2
miR-1
miR-133
miR-218
*
*
**
Relative miRNA expression level
*

Supplement: Additional file 2: Figure S2 — Differential expression levels of miR-1, miR-133a and miR-218 between normal cells and bladder cancer Expression of miR-1, miR-133a and miR-218 were detected in HUV-EC-C and L-02 cells. miRNA level in HUV-EC-C cells was regarded as standard and U6 was selected as endogenous reference. Means ± SEM of three independent experiments were shown. (PPT 115 kb) [file 1756-9966-32-10-S2.ppt]
